# Chronic pain in children and young people with cerebral palsy: a narrative review of challenges, advances, and future directions

**DOI:** 10.1186/s12916-024-03458-0

**Published:** 2024-06-11

**Authors:** Adrienne Harvey, Nadine Smith, Meredith Smith, Katarina Ostojic, Carolyn Berryman

**Affiliations:** 1 Neurodisability and Rehabilitation, Murdoch Children’s Research Institute, 50 Flemington Road, Parkville, VIC 3052 Australia; 2 Kids Rehab, Perth Children’s Hospital, 15 Hospital Avenue, Nedlands, WA 6009 Australia; 3 School of Allied Health Science and Practice, University of Adelaide, North Terrace, Adelaide, SA 5005 Australia; 4 Community Paediatrics Research Group, Sydney Medical School, The University of Sydney, Susan Wakil Health Building, Western Avenue, Camperdown, NSW 2050 Australia; 5 Innovation, IMPlementation and Clinical Translation (IIMPACT) in Health, University of South Australia, North Tce, Adelaide, South Australia 5001 Australia; 6 Hopwood Centre for Neurobiology, South Australian Medical Research Institute (SAHMRI), North Tce, Adelaide, South Australia 5005 Australia; 7 Paediatric Chronic Pain Service, Women’s and Children’s Hospital, King William Rd, North Adelaide, South Australia 5006 Australia

**Keywords:** Cerebral palsy, Pain, Chronic pain, Biopsychosocial model

## Abstract

**Background:**

Cerebral palsy (CP), the most common physical disability of childhood, is often accompanied by a range of comorbidities including pain. Pain is highly prevalent in children and young people with CP, yet has been poorly understood, inaccurately assessed, and inadequately managed in this vulnerable population. This narrative review presents recent research advances for understanding and managing pain in children and young people with CP, focusing on chronic pain, and highlights future research directions.

**Main body:**

Pain prevalence rates in CP vary due to different methodologies of studies. Recent systematic reviews report up to 85% of children experience pain; higher in older children, females, and those with dyskinesia and greater motor impairment. Research examining the lived experience perspectives of children and their families demonstrate that even those with mild motor impairments have pain, children want to self-report pain where possible to feel heard and believed, and management approaches should be individualized. Notably, many children with cognitive and communication impairments can self-report their pain if adjustments are provided and they are given a chance. Past inadequacies of pain assessment in CP relate to a focus on pain intensity and frequency with little focus on pain interference and coping, a lack of tools appropriate for the CP population, and an assumption that many children with cognitive and/or communication limitations are unable to self-report. Recent systematic reviews have identified the most reliable and valid assessment tools for assessing chronic pain. Many were not developed for people with CP and, in their current form, are not appropriate for the spectrum of physical, communication, and cognitive limitations seen. Recently, consensus and co-design in partnership with people with lived experience and clinicians have identified tools appropriate for use in CP considering the biopsychosocial framework. Modifications to tools are underway to ensure feasibility and applicability for the spectrum of abilities seen.

**Conclusion:**

Recent research advances have improved our understanding of the prevalence, characteristics and lived experience of chronic pain, and refined assessment methods in children and young people with CP. However, the very limited evidence for effective and novel management of chronic pain in this population is where research should now focus.

## Background

Cerebral palsy (CP) is the most common physical disability of childhood with a global prevalence rate of 1.6 per 1000 live births [1]. Pain is highly prevalent in children and young people with CP, with up to 76% experiencing any pain [2] and approximately one-in-three experiencing chronic pain [3]. There are multiple potential drivers of pain including hypertonia, musculoskeletal issues, and gastrointestinal concerns. Additionally, many children undergo multiple painful procedures and surgeries throughout childhood. Despite its high prevalence, pain has been poorly understood and inadequately assessed and managed in this vulnerable and heterogeneous population, resulting in reduced social and school engagement, quality of life, and psychological wellbeing [4]. At this present juncture when there is enthusiastic clinical and research interest in addressing chronic pain in children and young people with CP, a synthesis of current evidence for the prevalence, characteristics, assessment, management, and classification of pain in children and young people with CP is a critical step. This narrative review is informed by clinician researchers currently active in this field and presents the challenges and solutions for accurate assessment and management practices, as well as future research directions.

CP is an umbrella term that refers to a group of permanent disorders that affect movement and posture resulting from injury or insult to the developing brain {Rosenbaum, 2007 #19}. Individuals with CP have a considerably higher burden of medical, neurological, and mental/behavioural disorders compared with the general population, including those not directly related to the brain injury [5], and a range of associated impairments including visual, hearing, communication, and intellectual impairments, and also epilepsy [6]. Pain is the most common secondary condition in people with CP [7].

Pain is defined as ‘an unpleasant sensory and emotional experience associated with, or resembling that associated with, actual or potential tissue damage’ and chronic pain is pain that persists or recurs for longer than 3 months or beyond the expected time to heal [8]. Assessment and treatment using a biopsychosocial model is considered best practice to understand the multiple contributors to chronic pain in children, since the development and persistence of chronic pain involves the interaction of neurosensory (nociceptive), emotional, sociocultural, behavioural, and cognitive factors [9]. Until recently, this model was not applied to the CP population, resulting in sub-optimal care. Chronic pain contributes to interrupted sleep, diminished capacity to participate in activities, and reduced quality of life and health status of children and young people with CP [2, 10].

Children with severe intellectual disabilities, including those with CP, are more at risk of having pain but are rarely involved in research, causing an inequity in health care and benefit of being involved in research [11]. It is imperative that we are adaptable in our approaches to ensure equitable access and holistic outcomes for children with developmental disabilities such as CP who may have differing functional, communication, and cognitive abilities [12].

A narrative review structure [13] was chosen to synthesize recent pivotal changes in the classification of pain with a current understanding of the prevalence, assessment, and management of chronic pain in children and youth with CP. The review includes validation, empirical and experimental studies, and advocacy body reports. We included studies from the last 30 years, the majority of which (75%) were published within the last 10 years. The review is presented in the following order: the prevalence and lived experience of pain in children and young people with CP, the new classification of pain, assessment and management of chronic pain in children and young adults with CP, and suggestions for the future directions of the field.

## Prevalence and characteristics of pain in children and young people with CP

A systematic review of children and young adults with CP (57 studies) found large variations in rates of reported pain with prevalence between 14 and 76%, due to methodological issues with studies, sampling bias, inconsistent measurement, varying recall periods, and use of different participant age ranges [2]. More recent prospective cross-sectional studies have examined the prevalence and characteristics of pain in children and adolescents aged 5–18 years with CP using a range of parent and self-report outcomes [3, 14]. Acute pain was reported in 67% and chronic pain in 31% of 280 children; 42% of those with acute pain also reported chronic pain [3]. Pain was prevalent in 85% of 75 children with CP and dyskinesia; chronic in 77% [14]. Pain is more prevalent in females, non-ambulant children, children with dyskinesia, and older children [2, 3]. Pain often occurs at multiple body locations with the lower limbs, back, and abdomen most common [2]. Face, jaw, and temple pain is more common in children with dyskinesia, whilst non-ambulant children have more body sites affected than ambulant children [14]. Clinicians identify hip dislocation/subluxation, dystonia, and musculoskeletal deformity as common contributing factors to pain (15); however, more high-quality research with children and young people self-reporting their pain is required.

Several factors may contribute to a high prevalence of pain in children and young people with CP. Non-invasive management such as therapies and orthoses, as well as invasive management including surgeries and procedures such as botulinum toxin-A injections, are common throughout childhood [3, 14, 15]. The use of equipment such as standing frames and customized wheelchairs, need for feeding via nasogastric tubes or gastrostomy, and mobilization activities which include positioning, transferring, and walking are commonly associated with pain [16]. Mobility limitations experienced by many children and young people with CP increase the likelihood of chronic pain [3].

Hypertonia seen in spasticity and dystonia, particularly dystonia, also contributes to pain in children with CP [17, 18]. Secondary musculoskeletal impairments such as hip displacement, muscle contractures, scoliosis, and gastrointestinal dysfunction are commonly associated with pain [14, 17, 18].

## The lived experience of pain in CP: the perspectives of children and their caregivers

The perspectives of people with lived experience are vital to better understand their pain experience and to guide improvements in management specific to children and young people with CP. This is particularly pertinent for those with cognitive and/or communication limitations which may impact their ability to self-report their pain.

Three recent qualitative studies have explored the pain experiences of children with CP aged 8–18 years [15, 19, 20]. A study of 14 children with varying physical and cognitive abilities resulted in the main theme of ‘I have to obey my pain’ [15]. Emerging from the data was the varied pain experiences for children, the mental struggle involved, children having to make adjustments to manage their pain, and that being understood was the most important help [15]. Data from 10 children with CP found three superordinate themes: [1] Everybody’s experience of pain is different; [2] When the pain is winning; and [3] I know how to deal with it [20]. Pain interfered with school, physical activity, and psychosocial functioning and children described personalized strategies used to deal with pain [20]. Eight children with CP and dyskinesia reported that pain had been persistent throughout their childhood and was a usual part of their life, and that they were still learning strategies to manage their pain, including a sense that they needed to ‘push through’ their pain [19].

Caregivers also provide an important and unique perspective in two recent qualitative studies [21, 22]. Ten parents of children with dyskinetic CP reported the continual challenge of problem-solving pain, the pursuit of a solution, unfulfilled preferences for managing pain, the effects on families, and ongoing impacts with age [21]. Additionally, the perspectives of 14 parents of children with CP resulted in the overall theme ‘My child’s pain is just one piece of a complex jigsaw puzzle’ [22].

Key messages from qualitative studies involving children and young people with CP and their families are presented in Table 1.

**Table 1:** Key messages from the perspectives of people with lived experience

Lived experience perspectives
Pain is a determining feature of the lives of children with CP and their families [21]
Pain is a burden and impacts on family [20–22]
The pain experience is individualized, and others do not understand its complexity [15, 20]
Health professionals should prioritize understanding a child’s own pain descriptions as they want to feel heard and believed [15]
Pain management approaches should be tailored to individuals [15]
Pain education is required for children and families [15, 22]
Many children with cognitive and communication impairments can report their pain if adjustments are provided and they are given a chance [15, 19]

## Classifying pain in children with CP

Effective and appropriate management of pain relies on identifying and understanding underlying pain mechanisms (the biology, anatomy, and physiology of pain) and contributing factors from psychosocial domains. Consistent systematic classification of pain may benefit children with CP by promoting enhanced treatment choices and stimulating the development of novel interventions responsive to the child’s needs, e.g. novel pharmaceutical agents and behavioural interventions.

Classifying pain using the International Classification of Diseases—11 (ICD-11) has been proposed by authors of a review (195 original research papers) on pain in children with CP [23]. The ICD-11 differentiates between acute and chronic pain and further, chronic pain overall as chronic primary pain (pain cannot be explained by another condition) or chronic secondary pain (pain is a symptom of the underlying condition). Within these classifications are descriptors that relate to the underlying mechanisms that may contribute to a pain type: nociceptive pain when nociceptors have been activated by inflammation and tissue damage; neuropathic pain associated with nerve damage; and nociplastic pain which is thought to arise from altered nociception and augmented central nervous system (CNS) pain processing and is associated with other clinical symptoms derived from the CNS such as fatigue, gut dysfunction, and problems with sleep, memory, and concentration [24].

The review [23] reported the most common pain type in children with CP was musculoskeletal pain (41.5% of studies) and classified it under chronic secondary pain because of its association with other ‘causative’ factors such as muscle spasm, subluxation, scoliosis, osteoporosis, and joint misalignment. There are, however, inherent challenges involved in linking musculoskeletal pathology to symptoms and hence a more likely phenotype may typically have features of all three mechanisms and be classified as chronic primary pain. Acute pain was also reported and represented in 20% of studies, with only one study differentiating between acute and chronic pain. Acute procedural and post-surgical pain were the most common, and both were classified as secondary pain underpinned by nociceptive mechanisms.

Considering the phenotype of the presenting complaint is an alternative and arguably clinically more compelling way to classify pain in children with CP. The ICD-11 can be used to do this but a retrospective cohort study of data from a large insurance claims database for adults with CP and spina bifida grouped pain disorders into four pain types that included at least one mechanism described by the earlier ICD-9 [25]. The four pain types were (1) nociceptive pain (pain in limb, joint pain, etc.); (2) nociplastic pain (chronic pain, central pain syndrome, chronic pain syndrome, psychogenic pain, fibromyalgia, bladder pain syndrome, headache [including migraines], etc.); (3) neuropathic pain (e.g. neuralgia and neuritis); and (4) other/unspecified pain [25]. By recognizing the characteristics of pain types, health care professionals can establish a working hypothesis of the processes underpinning the problem and design an appropriate and effective intervention.

Indeed, quantitative sensory testing profiles for the detection of central pain mechanisms have been assessed in children and youth with CP revealing the presence of a combination of hyperalgesia and hypoesthesia [26]. Further work is needed, however, to interpret the results for clinical relevance. For example, combining these sensory testing methods with a neuropathic pain screening tool such as painDETECT (validated for use in adolescents) [27] and/or an impact of pain questionnaire which includes the report of other CNS-derived symptoms such as fatigue and gut dysfunction might provide a deeper understanding of the mechanisms underpinning the chronic pain presentation. A recent invited review of pharmacological management in CP [18] supported accurate identification of chronic pain mechanisms in CP as the way forward to enhanced chronic pain management. They cite evidence for the promise of hair cortisol levels as a biomarker for chronic pain in children and youth with CP [28], including children with communication difficulties. Future studies to test and validate methods that can characterize chronic pain mechanisms in children and youth with CP are needed.

## Assessment of pain in children and young people with CP

Inadequacies of pain assessment in children and young people with CP in the past relate to a previous focus on pain intensity and frequency, with little focus on pain interference or pain coping. This is despite the knowledge that maladaptive coping strategies such as helplessness, catastrophizing, and fear avoidance are associated with poorer functional outcomes [29, 30] and that children with CP show fewer positive coping strategies than typically developing children [31]. Pain intensity tells little about how pain is impacting/interfering with life. Additionally, a lack of tools appropriate for the range of communication, cognitive, and physical abilities seen in the CP population and an assumption that many children with CP are unable to self-report have contributed to inadequate pain assessment. Inconsistent pain assessment compromises effective pain management, potentially causing poorer quality of life and reduced participation [4].

Assessment tools can be categorized as (1) patient-reported outcome measures (PROMs), or self-report tools, or (2) observer-reported outcome measures (ObsROMs). ObsROMs are defined as observations made, appraised, and recorded by an individual other than the patient (e.g. proxy measures) [32]. Although self-report is always recommended due to the subjective nature of chronic pain, the ability of individuals with intellectual impairments to self-report may be limited or absent, depending on the severity of their condition [33]. In a study of 280 children aged 5–18 years with CP, 36% were unable to self-report due to intellectual or communication impairment, whilst 46% could self-report. However, only children over 9 years of age had the option to self-report due to psychometric properties of outcome measures chosen [3].

A modified Delphi study of 83 clinicians and people with lived experience identified 12 essential domains for measuring chronic pain in CP: pain location, frequency and intensity, changeable factors, impact on emotional wellbeing, impact on participation, pain communication, impact on quality of life, physical impacts, sleep, pain duration, and pain expression [34]. These now guide assessment tool selection specific to CP.

The Holland Bloorview Chronic Pain Assessment Toolbox for Children with Disabilities, which was informed by an earlier systematic review [35, 36], was instrumental in initially collating and assessing chronic pain tools for CP. This toolbox, more recent systematic reviews, and other reviews have identified recommended tools for assessing chronic pain in children with CP [12, 37–41]. The combined literature highlights the lack of use and validation of pain coping tools in the CP population. Table 2 displays the recommended tools that focus on chronic pain interference and coping, grouped into PROMs (child self-report) and ObsROMs (parent proxy report or observational tools). Although many tools were not developed for people with CP and, in their current form, may not be appropriate for the spectrum of physical, communication, and cognitive limitations seen in this population, many have potential if modified. Children with complex communication needs, for example augmentative and alternative communication users, or children with cognitive or significant physical limitations require modified tools to ensure they can self-report wherever possible.

**Table 2:** Recommended assessment tools for assessment of chronic pain interference or coping in children and young people with cerebral palsy. Tools with content validity in CP are indicated by*

Tools	Pain interference	Pain coping
**PROMs**	Bath Adolescent Pain Questionnaire [42] Child Activity Limitations Interview [43] Pain Interference Index [44] Modified Brief Pain Inventory [45] Patient-Reported Outcome Measurement Information System [46]	Bath Adolescent Pain Questionnaire [42] Cerebral Palsy Quality of Life-teen* [47] Child Self-Efficacy Scale [48] Fear of Pain Questionnaire for children [49] Fear of Pain Questionnaire for children—short form [50] Pain Catastrophizing Scale [51] Pain Coping Questionnaire [52] Pain Coping Questionnaire short form [53] Pediatric Pain Coping Inventory [54]
**ObsROMs**		
Parent proxy	Patient-Reported Outcome Measurement Information System Pediatric Proxy Pain Interference Scale [46] Bath Adolescent Pain Questionnaire for Parents [55] Modified Brief Pain Inventory-Proxy [45]	Fear of Pain Questionnaire parent version [49] Pain Catastrophizing Scale [51] Pain Coping Questionnaire parent version [52] Pediatric Pain Coping Inventory parent version [54]
Observational tools	Paediatric Pain Profile* [56] The Non-communicating Children’s Pain Checklist—Revised* [57]	Not appropriate

*PROMs* Patient-reported outcome measures, *ObsROMs* Observer-reported outcome measures

*Indicates that these tools have content validity in cerebral palsy

There is some disagreement about the most reliable, valid, and feasible pain interference tools for use in CP as the relevance of specific items to an individual is often impacted by their age, mobility level, and cognitive ability. To date, most pain assessment tools were not designed or validated with children with CP. Thus, they were often validated in general paediatric cohorts where children were verbal communicators with no intellectual disability and no physical disability. Currently, recommended tools for self-reporting pain interference are the Bath Adolescent Pain Questionnaire [42], the Child Activity Limitations Interview [43], the Modified Brief Pain Inventory [45], and the Patient-Reported Outcome Measurement Information System [46]. However, none of these has been fully validated in children with CP. The Paediatric Pain Profile [56] is the most reliable and valid tool for children and adolescents unable to self-report pain interference and has been validated in CP. Other recommended ObsROMs for pain interference are listed in Table 1. Several tools are available to assess pain coping; the most feasible for young people with CP are the Fear of Pain Questionnaire [49], the Fear of Pain Questionnaire short-form [50], and the Pain Coping Questionnaire short-form [53]. Although many tools have proxy as well as self-report versions, discrepancies between self- and proxy-report have been noted when assessing the impact of pain on emotional functioning and interference [58]. This may in part be due to caregiver’s mental and physical health potentially influencing their proxy-report perspective, with poorer caregiver mental health associated with higher parent/carer proxy scores [59]. Caregivers have also identified that given the personal and subjective nature of pain, it is very difficult to estimate the impact of their child’s pain [22]. This is even more difficult for parents of children with cognitive and communication impairment.

Whilst it is important to prioritize self-report where possible, particularly for pain coping, there is a need for flexibility when eliciting self-report from children and young people with cognitive impairment. The decision as to whether a person’s responses are accurate is somewhat subjective, even with cognitive screening. In research settings, it may be appropriate to do a cognitive screening activity, but in clinical settings some self-report (regardless of the perceived accuracy) is still valuable. If parents/caregivers or health professionals are concerned about the accuracy of self-report and the decision is made to use ObsROMs (either to complement or instead of self-report), it has been suggested that strategies such as using multiple observers, including parents, may provide more accurate information [12]. Most self-report tools expect a cognitive ability of 8 years of age which precludes many people in the CP population from accessing self-report. If the response scale of a tool is not well understood, it could be modified rather than assuming a person is not capable of self-report. Our team has been exploring alternative administration methods, for example Talking Mats (www.talkingmats.com), which are known to reduce cognitive load and improve understanding, to obtain self-report more accurately from this group. Considering that 51% of children with CP have no reported intellectual disability and 37% have no reported speech impairment [6], many children will have the communication and intellectual abilities to self-report when outcome measures have been modified with minor modification to tool. Other children will require more extensive modifications to pain assessment tools.

Recent research using focus groups, workshops, and Delphi processes with clinicians and people with lived experience have refined the list of tools to the few appropriate in their current format, and those requiring modifications to ensure accessibility for children and young people with CP. Co-design methods, in partnership with young people with CP, parents of children with CP, and clinicians, have gained consensus on modifications to tools and mapped tools to essential domains using a biopsychosocial framework [60]. These detailed modifications (and subsequent validations studies) will help ensure that children understand tool instructions, the magnitude of the scales, and any pictorials included within tools.

## Management of pain in children and young people with CP

There is a paucity of high-quality research for effective management of chronic pain in children and young people with CP. A systematic review examining the efficacy of interventions in children and adolescents with CP (57 studies) found many studies were low-moderate quality [10]. There is moderate-high evidence to support intrathecal baclofen therapy for pain secondary to hypertonia in spastic and spastic-dyskinetic CP, non-pharmacological interventions (including distraction and biofeedback) for procedural pain, and pharmacological interventions for postoperative pain [10, 61]. Low-quality evidence exists for deep brain stimulation to reduce pain severity, frequency, and analgesia requirement in children with severe dystonic CP [62]. However, some of these interventions are highly specific and unlikely to be suitable for the majority of young people with CP, particularly those with chronic pain. A pilot feasibility study of gabapentin for managing chronic pain in 13 children with dystonic CP suggests gabapentin may improve pain behaviour, care, and comfort and attainment of pain-related goals [63]; however, further research is required to confirm these findings.

Pain is often associated with interventions used for the management of CP [10]. Pain during and after procedures is common; however, there is a slow uptake of the application of evidence-based, non-pharmacological management in this setting. Research indicates a role for distraction, imagery, and preparation/education for children and young people with CP [10, 61].

Activity is an evidence-based management strategy for many chronic health conditions, including chronic pain [64]; however, the evidence for effect in children with CP and chronic pain, many of whom have considerable mobility limitations, is unknown. A single subject research design study using individualized and tailored movement interventions for 8 weeks in three non-ambulant children with dyskinetic CP found evidence of parent-reported improvements in their child’s pain and care and comfort [65].

In the face of limited evidence to guide chronic pain management for children, families, and clinicians, parents and children report using positioning, stretching, massage, heat/cold, rest, controlled breathing, short-acting analgesics, and hydrotherapy for pain management [3, 19]. Parents are requesting interventions other than pharmacological as many children take numerous medications which are often limited by adverse side effects [21]. Clinicians have reported key barriers to effective pain management including a lack of access to some interdisciplinary team members and inadequate pain education for health professionals and children and families [66, 67].

Non-pharmacological and interdisciplinary strategies are necessary for chronic pain management in children and young people with CP. Internet-delivered family cognitive behavioural therapy should also be considered, and existing online tools tailored to meet the spectrum of abilities within the CP population [10].

## Discussion and future research directions

This narrative review provides an overview of the current knowledge base for chronic pain in children and young people with CP. Pain is highly prevalent and presents several challenges in this heterogeneous population. The range of mobility, communication, and cognitive abilities seen in CP impacts on how pain presents, how we can accurately assess it, and how we tailor effective management. In addition, the ability of many children and young people with CP to self-report their pain may be limited to varying degrees, further challenging our assessment and management.

Recent advances have increased our understanding of the lived experience of chronic pain in children and young people with CP and ways to understand the underlying mechanisms of chronic pain in CP are emerging. Significant improvements in how we identify and assess pain using a biopsychosocial approach have been achieved ensuring inclusion of pain coping and alternative methods of administration of tools to increase inclusion for those with communication and cognitive limitations.

Despite the high prevalence and numerous potential contributors, there is limited high-quality evidence clearly confirming the main contributors to pain, particularly chronic pain, in children and young people with CP. Future research should focus on improving our understanding of the underlying mechanisms of chronic pain in this population so that we can develop more effective management strategies. Whilst previous research has concentrated primarily on nociceptive pain, the focus should now shift to a deeper understanding of other pain mechanisms including neuropathic and nociplastic mechanisms of pain.

The lack of high-quality research for effective management of chronic pain in children and young people with CP necessitates an urgent need to move forwards in this area, whilst still making gains in understanding the underlying mechanisms and refining our assessment. We have viable methods to assess and document the efficacy of management strategies for chronic pain available now, and we know that interdisciplinary interventions are the gold-standard for chronic pain management in primary pain conditions and disease-related pain [68]. We now need to implement these in CP. Children and young people with CP already receive interdisciplinary care to manage their primary physical disability; therefore, the gold-standard approach for pain management closely aligns with this. Further research is needed to explore tailored interdisciplinary interventions adapted to suit the varying specialized needs and abilities of children with CP and chronic pain, with activity and psychological interventions as key components. A focus on improving active pain coping strategies for children and young people with CP is also a priority.

## Conclusions

Chronic pain is highly prevalent and disruptive to everyday functioning in children and young people with CP. A biopsychosocial approach is imperative to effectively assess the impact chronic pain has on a range of domains including emotional functioning, and alternative methods are required to ensure children with complex communication needs can self-report wherever possible. With a deeper understanding of the mechanisms of pain in children and young people with CP, effective and interdisciplinary approaches can be designed and implemented to ensure children and their families can participate fully in their everyday lives.

## Data Availability

Data sharing is not applicable to this article as no datasets were generated or analysed during the current study.

## Abbreviations

CCN: Complex communication needs
CP: Cerebral palsy
ICD: International Classification of Diseases
ObsROMs: Observer-reported outcome measures
PROMs: Patient-reported outcome measures
